# A Baseball Suture Technique With Single Bone Tunnel and Button Fixation for Flexor Digitorum Profundus Avulsion Repair

**DOI:** 10.7759/cureus.110953

**Published:** 2026-06-16

**Authors:** Zoe K Papadopoulou, Vasilios Raoulis, Konstantinos N Malizos, Alexis T Kermanidis, Aristidis H Zibis

**Affiliations:** 1 Department of Anatomy, Faculty of Medicine, University of Thessaly, Larissa, GRC; 2 Department of Mechanical Engineering, School of Engineering, University of Thessaly, Volos, GRC

**Keywords:** baseball suture, distal phalanx, fdp avulsion, flexor digitorum profundus, flexor tendon repair, hand surgery, jersey finger, looped suture, pullout button fixation, transosseous tunnel

## Abstract

Flexor digitorum profundus (FDP) avulsion injuries require secure tendon fixation within the limited footprint of the distal phalanx to achieve strong tendon purchase while minimizing tendon trauma, while osseous compromise remains a surgical challenge. We propose a novel surgical technique for FDP avulsion repair using a baseball suture configuration, a single transosseous bone tunnel, and dorsal pullout button fixation. The tendon is secured with a multi-pass baseball suture technique and advanced through a single bone tunnel in the distal phalanx, thereby providing stable fixation while minimizing osseous disruption. A protective interface is interposed beneath the dorsal button to reduce pressure-related complications at the nail apparatus. This approach is designed to improve tendon purchase while reducing tendon penetrations and minimizing osseous disruption. The accompanying images are derived from a cadaveric specimen to enhance visualization of the described technique. This method represents a simple and reproducible option for FDP avulsion repair, offering potential mechanical and practical advantages. Further biomechanical and clinical studies are required to validate its effectiveness.

## Introduction

Flexor digitorum profundus (FDP) avulsion injuries are uncommon but functionally significant lesions that require stable reinsertion to restore distal interphalangeal joint flexion [1,2]. Flexor digitorum profundus avulsion injuries, commonly referred to as “jersey finger” injuries, typically occur following forced extension of a flexed distal interphalangeal joint, resulting in avulsion of the tendon from its insertion on the distal phalanx. Because the FDP tendon is the sole flexor of the distal interphalangeal joint, untreated injuries may result in substantial impairment of finger flexion and hand function [1,2]. Several repair methods have been described, including pullout sutures tied over a dorsal button, suture anchors, and transosseous fixation techniques [2-5]. Each method presents specific advantages and limitations related to fixation strength, technical complexity, implant cost, and potential complications involving the nail unit or distal phalanx [4-6].

Secure tendon purchase is a critical determinant of repair reliability, especially in distal tendon avulsion patterns with limited residual tendon length or compromised tissue quality [7]. Looped suture constructs, originally developed for ligament reconstruction, have demonstrated favorable tensile properties and load distribution characteristics. These features may be advantageous in FDP avulsion repair, where secure tendon purchase and resistance to suture pull-through are critical considerations. However, their use in FDP avulsion repair has not been widely emphasized.

We describe a technical modification for FDP avulsion repair using a baseball suture configuration combined with a single transosseous bone tunnel and dorsal pullout button fixation. The tendon is secured with a multi-pass baseball suture construct and advanced through a single tunnel in the distal phalanx, allowing secure fixation while minimizing osseous disruption. A protective interface is interposed beneath the dorsal button to reduce pressure-related trauma to the nail apparatus. This technical report outlines the surgical steps, underlying rationale, and key practical and biomechanical aspects of the described technique.

## Technical report

Surgical technique

The patient is positioned supine with the affected upper limb placed on a hand table in supination. A pneumatic tourniquet is applied to the upper arm and inflated to an appropriate pressure, typically around 250 mmHg in our practice, to provide a bloodless field. Tourniquet pressure should be adjusted according to patient-specific factors and limb occlusion pressure.

A standard volar approach to the distal phalanx is performed, allowing identification of the FDP tendon and its insertion site. The avulsed tendon is isolated and prepared by debriding nonviable tissue to obtain an appropriate tendon end for repair.

**Figure 1:**
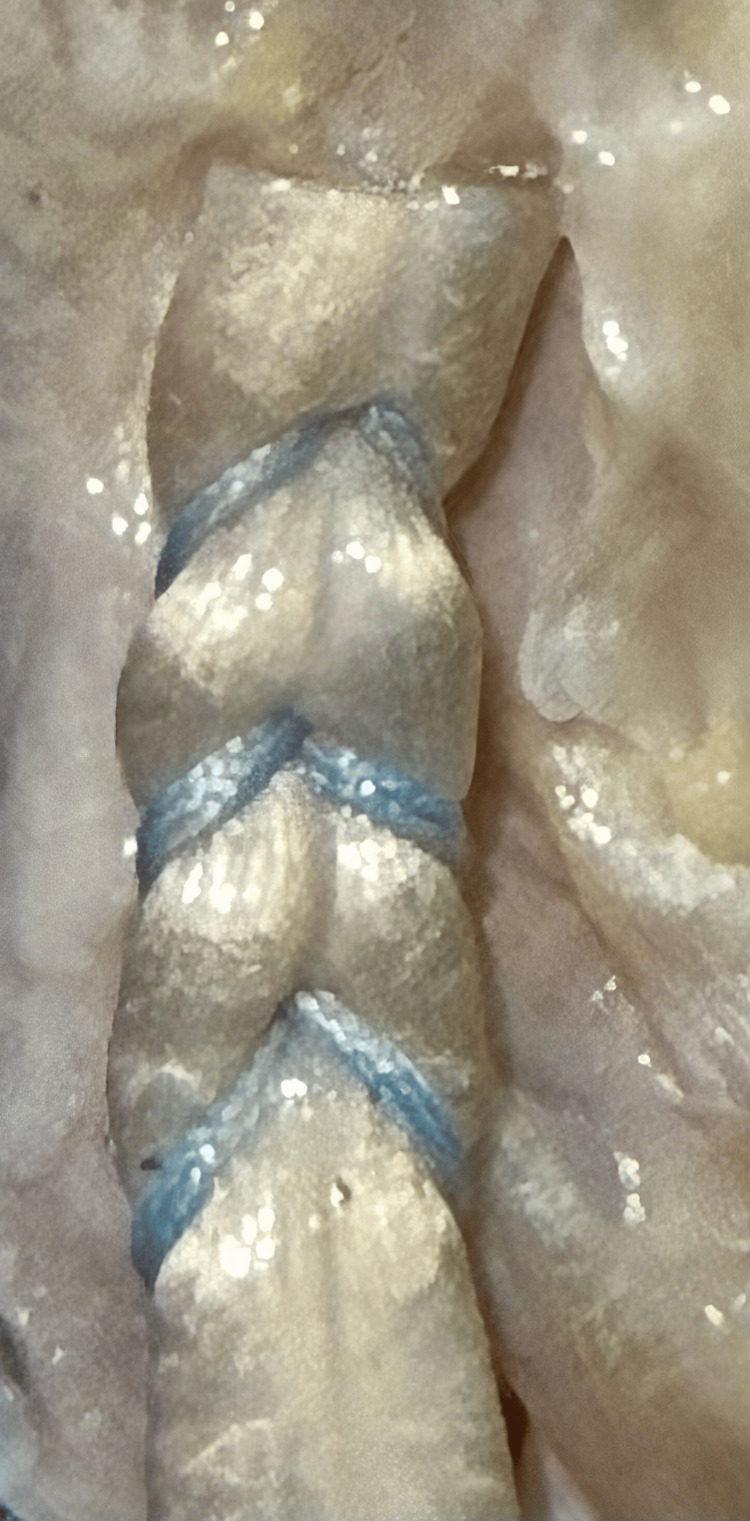
Multi-pass baseball suture configuration using looped suture Multi-pass baseball suture configuration using a looped nonabsorbable suture, demonstrating sequential tendon engagement and secure suture purchase.

A nonabsorbable No. 2 looped polyester suture mounted on a 75-mm straight needle (Wilsuture®, Péters Surgical, Bobigny, France) is used to secure the tendon with a multi-pass baseball suture configuration (Figure 1).

**Figure 2:**
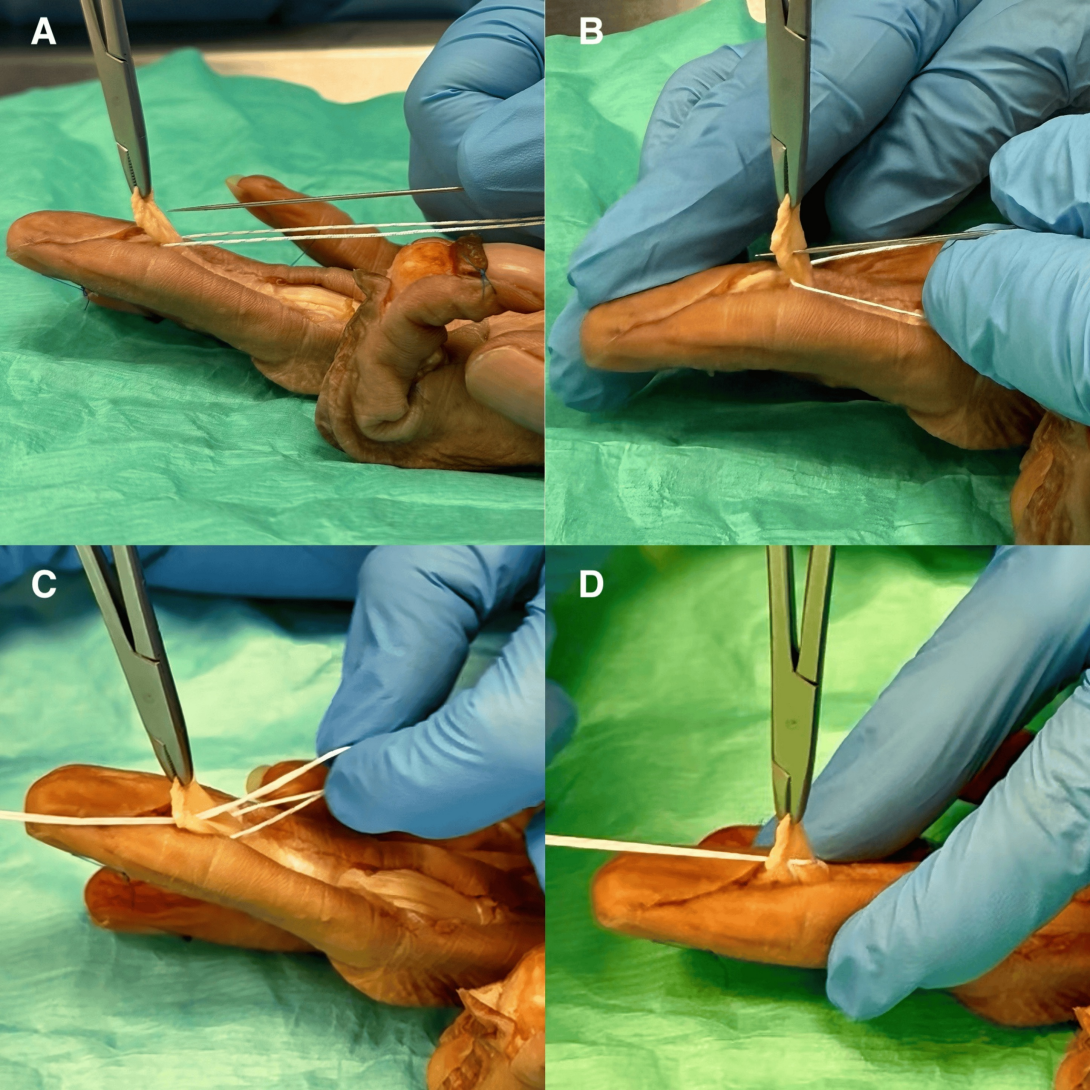
Stepwise tendon suturing technique using looped suture Stepwise demonstration of the multi-pass baseball suture technique using a looped nonabsorbable suture. (A–D) Sequential needle passes illustrating progressive tendon engagement and formation of the suture configuration.

The looped construct allows each passage to capture a broader segment of tendon tissue, achieving secure fixation while minimizing the number of tendon penetrations. Sequential passes are performed in a running, locking manner along the distal tendon stump (Figures 2A-2D) to create a stable construct, taking care to distribute the suture evenly and avoid excessive bunching.

**Figure 3:**
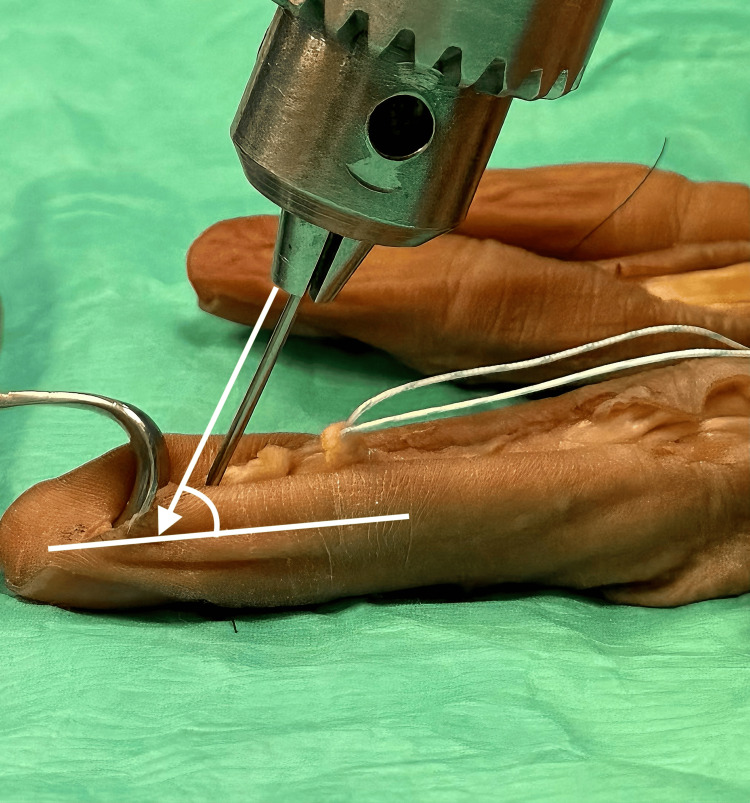
Transosseous tunnel creation using drill and needle Creation of a transosseous tunnel through the distal phalanx using an 18-gauge needle mounted on a powered drill. The tunnel follows a slightly oblique volar-to-dorsal trajectory directed distally. The dorsal exit point is created through the nail plate distal to the lunula, avoiding injury to the germinal matrix.

A single transosseous tunnel is created through the distal phalanx from the FDP footprint at the volar base of the distal phalanx toward the dorsal nail plate using an 18-gauge needle mounted on a powered drill. The tunnel follows a slightly oblique distal trajectory, with the dorsal exit point located through the nail plate distal to the lunula to avoid injury to the germinal matrix (Figure 3).

**Figure 4:**
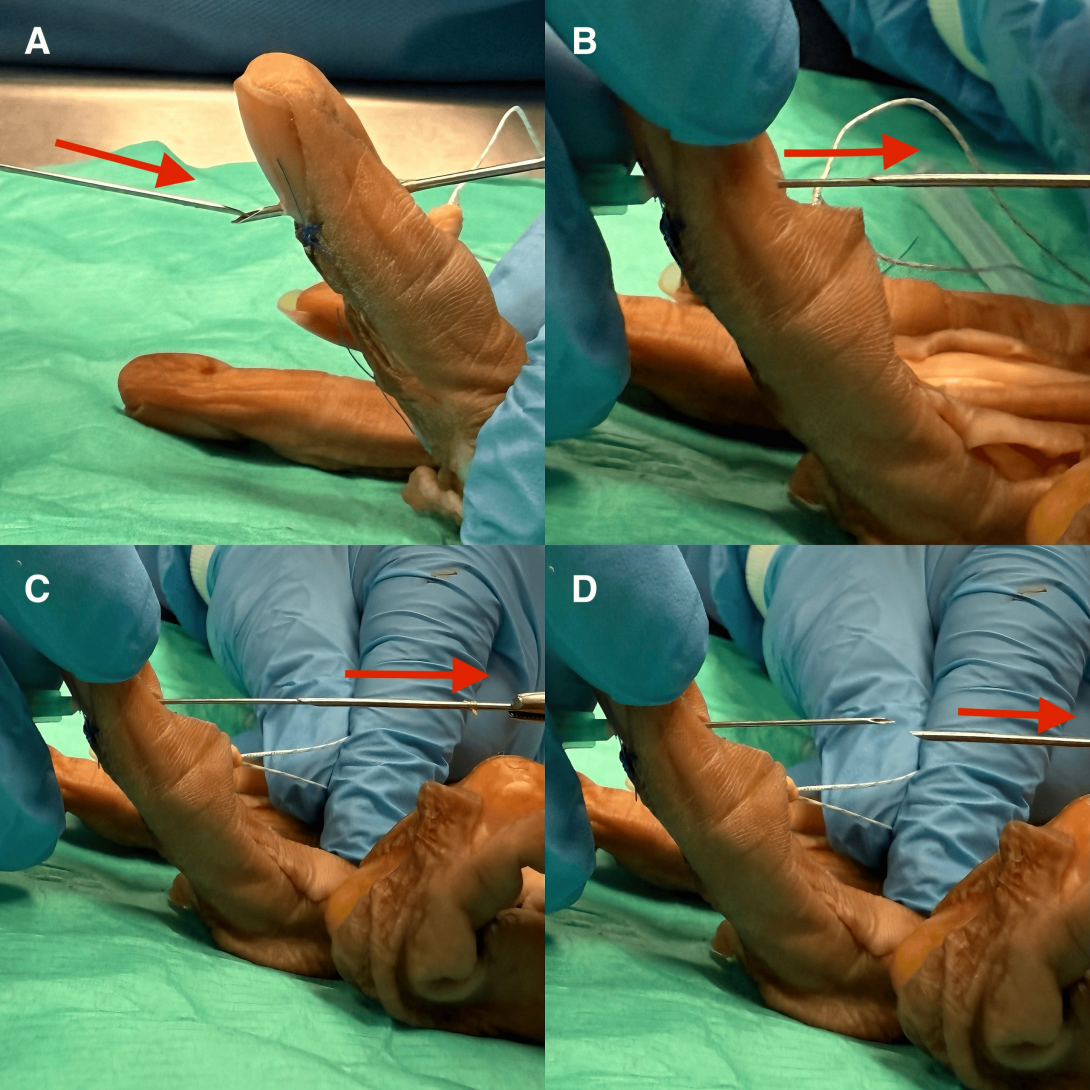
Needle exchange technique for transosseous tunnel guidance Needle exchange technique. (A–D) A second needle is introduced through the lumen of the initial needle and advanced in a dorsal-to-volar direction. The initial needle is then removed, leaving the finer needle in situ to preserve the tunnel trajectory.

After the needle exits dorsally, it is left in place to serve as a guide. Through the lumen of this needle, a second needle (21-gauge) is advanced in a dorsal-to-volar direction, effectively replacing the first needle while maintaining the established tunnel trajectory. The initial needle is then withdrawn (Figures 4A-4D).

**Figure 5:**
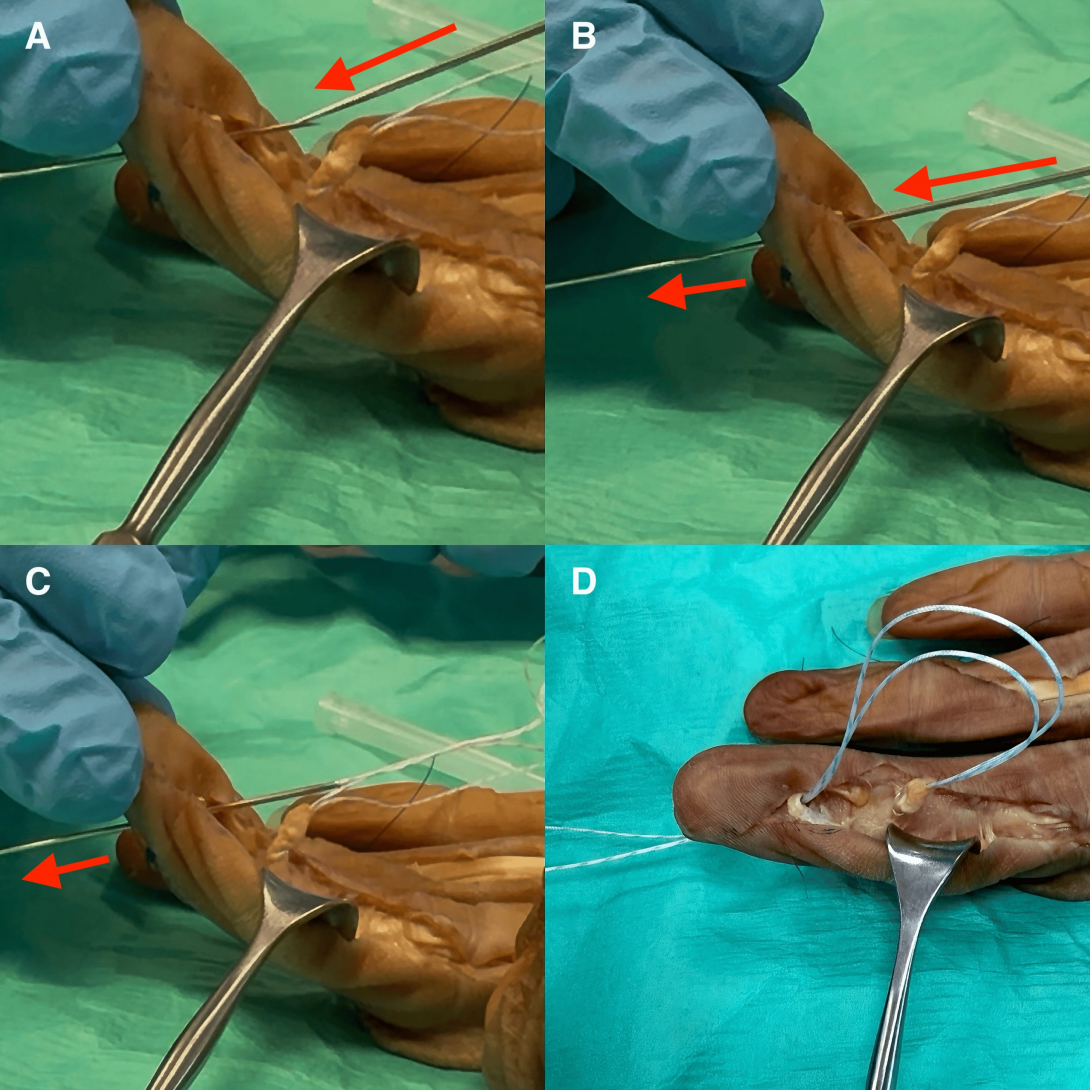
Suture passage through the transosseous tunnel Suture passage through the transosseous tunnel. (A–D) The suture needle is passed through the lumen of the guiding needle, facilitating controlled transosseous passage and dorsal delivery of the suture.

Subsequently, the suture is passed through the lumen of the second needle and delivered to the dorsal aspect of the finger, allowing controlled and atraumatic passage through the bone tunnel (Figure 5).

**Figure 6:**
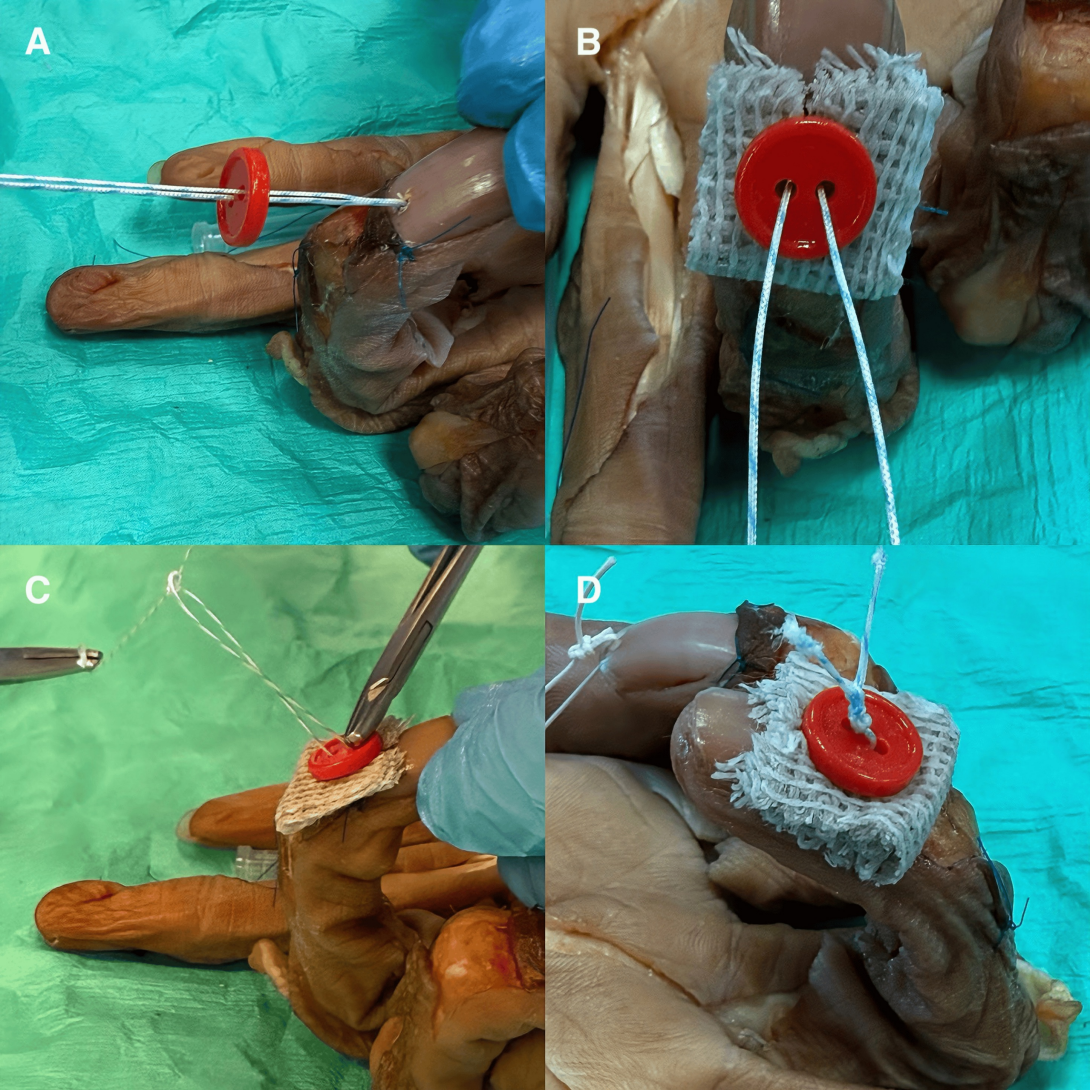
Final fixation using a dorsal pullout button Final fixation using a dorsal pullout button. (A–D) The suture ends are passed through the button and tied over a petrolatum gauze interface to protect the nail bed and underlying nail apparatus. The distal interphalangeal joint is maintained in flexion during tensioning to reduce stress at the repair site and enhance tendon-to-bone apposition.

The suture ends are secured over a dorsal pullout button. During fixation, the distal interphalangeal joint is maintained in flexion to reduce tension at the repair site and optimize tendon-to-bone contact. A petrolatum gauze interface is interposed between the button and the dorsal skin, according to the authors' routine practice, to minimize pressure-related injury to the nail apparatus (Figure 6).

The final construct is assessed for stability and appropriate tendon positioning at the distal phalanx footprint. For demonstration purposes, the accompanying images were obtained from a cadaveric specimen to enhance visualization of the described technique.

The key steps of the technique, including tendon suturing, transosseous tunnel creation, needle exchange, and final fixation, are demonstrated in the accompanying video (Video 1).

**Video 1:** Step-by-step demonstration of the surgical technique This video (duration: 1 min 11 sec; resolution: 1272 × 1280 pixels) demonstrates the sequential steps of the described technique, including multi-pass baseball tendon suturing, transosseous tunnel creation, needle exchange, suture passage, and fixation over a dorsal pullout button. No audio narration is included.

The images and video were obtained from cadaveric specimens and are presented solely for educational and technical demonstration purposes; no clinical patient data or identifiable patient information are included.

Postoperative rehabilitation

The following postoperative rehabilitation recommendations are based on commonly accepted flexor tendon rehabilitation protocols reported in the literature. Τhe finger is immobilized using a dorsal blocking splint, with the wrist positioned in neutral or approximately 20-30° of extension, the metacarpophalangeal joints in 50-70° of flexion, and the interphalangeal joints maintained in relative flexion to protect the repair. Early controlled mobilization is initiated within the first postoperative days, following established flexor tendon rehabilitation protocols such as the Duran or modified Kleinert regimens. This typically includes passive flexion exercises combined with controlled active motion within the limits of the splint. The splint is maintained for approximately 4-6 weeks, after which gradual progression to active motion is allowed. Forceful gripping and resisted flexion are avoided until approximately 8-10 weeks postoperatively to minimize the risk of repair failure [8-11].

## Discussion

FDP avulsion injuries remain challenging to manage due to the need for secure tendon fixation within the limited footprint of the distal phalanx [1,2]. Multiple techniques have been described, including pullout button fixation, suture anchors, and transosseous repairs, each with distinct advantages and limitations related to fixation strength, technical complexity, implant cost, and complication profile [2-5].

The technique presented herein combines a baseball suture configuration with a looped, nonabsorbable suture and single transosseous tunnel fixation, aiming to optimize tendon purchase while minimizing local tissue trauma and osseous compromise. The biomechanical rationale presented in this discussion is based on the design characteristics of the construct and established principles of tendon repair. While these concepts were not directly evaluated in the present study, they may help explain the potential behavior of the repair construct and merit further biomechanical investigation.

A key feature of this construct is the use of a looped suture, which allows effective tendon grasp with fewer individual passes through the tendon. By reducing the number of needle penetrations, this approach may reduce cumulative tendon trauma and the risk of suture pull-through, particularly in cases with a short or attenuated distal tendon stump [6,7]. Similarly, fewer tendon penetrations may help preserve tendon integrity and local vascularity while limiting excessive suture bulk within the repair site. Furthermore, the looped configuration may facilitate more uniform load distribution along the repair construct, potentially reducing focal stress concentration and excessive tendon strangulation [6,7,12].

The multi-pass baseball suture configuration provides broad circumferential engagement of tendon fibers and may improve load sharing across the repair construct by distributing forces over a larger tendon surface area. Consequently, this configuration may improve resistance to gap formation while maintaining a balance between secure fixation and avoidance of excessive focal compression of the tendon tissue, which has been associated with compromised vascularity and weakening of tendon tissue [7,12]. However, these proposed biomechanical advantages were not directly evaluated in the present study and require further biomechanical investigation.

The use of a nonabsorbable suture may contribute to construct robustness, as larger-caliber sutures have generally been associated with increased tensile strength and improved resistance to failure. In addition, looped suture constructs have been utilized in procedures requiring high fixation strength, indirectly supporting their potential applicability in tendon repair settings where secure tendon fixation is desired [12].

From an osseous standpoint, the use of a single transosseous tunnel may represent an advantage over techniques requiring multiple drill holes or implant insertion. By limiting bone violation to a single tunnel, the risk of iatrogenic weakening or fracture of the distal phalanx may be reduced, particularly in small or osteopenic bones [3,13]. However, this potential benefit was not specifically evaluated in the present study.

Compared with suture anchor-based techniques, the described method may also offer practical advantages in terms of cost and accessibility, as it relies on commonly available suture material and avoids the need for implants [4,14]. These characteristics may be particularly relevant in resource-limited settings or in situations where implant use is not feasible.

The use of a dorsal pullout button remains a simple and widely used method for distal fixation but carries a known risk of complications involving the nail unit, particularly when the dorsal exit point is improperly positioned relative to the germinal matrix [5]. In the present technique, the transosseous tunnel exits through the nail plate distal to the lunula in an effort to avoid injury to the germinal matrix. In addition, a petrolatum-gauze interface is interposed beneath the button to reduce direct pressure on the dorsal soft tissues and nail apparatus. Although the protective effect of these measures was not formally evaluated, they represent practical modifications intended to minimize nail-related and pressure-related complications.

Limitations

This study has several limitations. As a technical report, the manuscript is intended to describe the surgical technique and does not include clinical or functional outcome data. In addition, no biomechanical testing was performed to quantify fixation strength, assess gap formation, evaluate cyclic loading performance, or compare the construct with established repair techniques. Tendon gliding characteristics and potential effects on tendon excursion were not evaluated. Furthermore, no assessment of nail-unit complications or other procedure-related clinical outcomes was possible within the scope of this report. The proposed biomechanical and clinical advantages of the technique, therefore, remain to be confirmed. Future studies, including comparative biomechanical testing, cyclic loading analysis, and prospective clinical evaluation, are necessary to further validate the effectiveness and safety of this approach [15,16].

Despite these limitations, the described technique represents a simple and reproducible option for FDP avulsion repair. Its design aims to maximize tendon purchase while minimizing tendon and osseous disruption; however, the proposed biomechanical and clinical advantages of the construct require confirmation through future comparative biomechanical and clinical studies.

## Conclusions

The described technique represents a technically feasible and reproducible option for FDP avulsion repair, combining a baseball suture configuration with a looped nonabsorbable suture and single transosseous tunnel fixation. Its design aims to optimize tendon purchase while minimizing tendon and osseous disruption.

The potential biomechanical and clinical advantages of this construct were not directly evaluated in the present study. Further biomechanical and clinical investigations are warranted to validate its effectiveness, safety, and potential role in the management of FDP avulsion injuries.
